# Ecological environmental management program promotes *Schistosomiasis* control in Erhai Lake of China: an analysis based on SWOT-ANP-ADAM approach

**DOI:** 10.1186/s40249-026-01442-9

**Published:** 2026-04-13

**Authors:** Hongqiong Wang, Jing Song, Shizhu Li, Xinping Shi, Siqi Ning, Yuwan Hao, Chunhong Du, Yi Dong

**Affiliations:** 1 Department of Schistosomiasis Control and Prevention, Yunnan Institute of Endemic Disease Control and Prevention, 1181 Xianghe Road, Kunming, 650500 China; 2 Yunnan Key Laboratory of Natural Focus Disease Control Technology, Dali, 671000 China; 3 National Institute of Parasitic Diseases, Chinese Center for Disease Control and Prevention (Chinese Center for Tropical Diseases Research); National Key Laboratory of Intelligent Tracking and Forecasting for Infectious Diseases; Key Laboratory On Parasite and Vector Biology, Ministry of Health; WHO Centre for Tropical Diseases; National Center for International Research on Tropical Diseases, Ministry of Science and Technology, Shanghai, 200025 China; 4 School of Global Health, Chinese Center for Tropical Diseases Research-Shanghai Jiao Tong University School of Medicine, Shanghai, 200025 China; 5 School of Public Health, Kunming Medical University, Kunming, 650500 China; 6 School of Public Health, Dali University, Dali, 671000 China

**Keywords:** Schistosomiasis control, *Schistosoma japonicum*, Ecological environmental management, Lake governance, Erhai lake, SWOT-ANP-ADAM approach

## Abstract

**Background:**

The transmission of *Schistosoma japonicum* is closely related to the surrounding natural environment and socio-economic factors. In recent years, the ecological environmental management program has been implemented in *S. japonicum* endemic areas around Erhai Lake of China. Relevant protection and governance measures affected the transmission of *S. japonicum*. This study was conducted to assess the impact of ecological environmental management program on *S. japonicum* control, and proposed strategic alternatives with a prioritized order of implementation in Erhai Lake.

**Methods:**

An integrated SWOT-ANP-ADAM analysis is performed to accomplish the set objective in this study. The strengths, weaknesses, opportunities, and threats (SWOT) analysis is conducted to identify the impact factors of ecological environmental management program on *S. japonicum* control and strategic alternatives. The Analytical Network Process (ANP) was used to evaluate the impact factors, and the Axial Distance-Based Aggregated Measurement (ADAM) method was applied to constructed multi-faceted polyhedron for the ranking the strategic alternatives, thereby better informing decisions for synergistic ecological-disease management.

**Results:**

A total of 14 impact factors and 12 strategic alternatives were obtained. The relative importance of the group of impact factors is ranked as strengths, opportunities, weaknesses, and threats, with weights equal to 0.3092, 0.2610, 0.2324, and 0.1975, respectively. The Chinese central government's prioritization of *S. japonicum* control and ecological environmental management (weight equal to 0.1562), as well as the lack of a top-level cooperative mechanism designed to integrate *S. japonicum* control and ecological environmental management (weight equal to 0.1424), are the most noteworthy factors. In ecological environmental management program of Erhai Lake, management of agricultural non-point source pollution (weight equal to 0.1209), construction of high-efficiency water-saving irrigation systems(weight equal to 0.0853) reduce the risk of *S. japonicum* transmission, while the wetland restoration may create more favorable habitats for *Oncomelania hupensis*, as well as increase the number of wildlife populations posing challenges for *S. japonicum* control(weight equal to 0.0700). Additionally, construction of the ecological management- *S. japonicum* control model in an integrated manner is the most important strategy (volume of complex polyhedron equal to 0.0693).

**Conclusions:**

Ecological environmental management program in the Erhai Lake has significant strengths and opportunities in promoting *S. japonicum* control, but also faces certain weaknesses and threats according to the weight of impact factors. This study confirms ecological environmental management as a viable complementary strategy to conventional *S. japonicum* control. Scientific planning and comprehensive integration can maximize synergies between schistosomiasis control and ecological protection. Additionally, 12 strategic alternatives with prioritized implementation may provide suggestions for decision makers in similar areas to adopt strategic decisions, which are of great practical significance and application value.

**Graphical Abstract:**

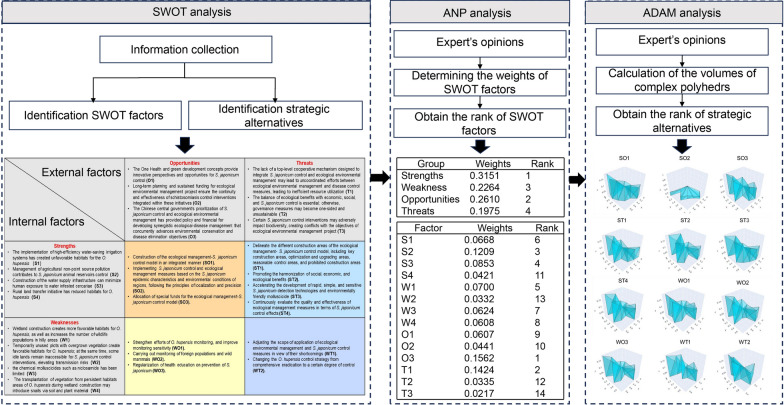

**Supplementary Information:**

The online version contains supplementary material available at 10.1186/s40249-026-01442-9.

## Background

Schistosomiasis is a neglected tropical parasitic disease that has posed a considerable burden affecting 240 million people worldwide, with more than 779 million people living in endemic areas [1]. The infection is predominantly endemic in tropical and subtropical regions, especially in poor communities without potable water and adequate sanitation [2]. It is a disease endemic to China, where the causative agent is *Schistosoma japonicum*. The disease reservoirs include humans, livestock, and wild mammals, while *Oncomelania hupensis*serves as the intermediate host snails [3, 4].

After infection with *S. japonicum*, the adult worms will parasitize the portal system to mate and lay eggs. Some eggs are excreted in the feces to continue the life cycle of the parasitic disease, while others remain in host tissues, triggering an immune response that gradually damage the liver and intestinal lining, and other organs. This pathology results in clinical symptoms such as abdominal pain, diarrhea, and blood in the stools [5]. After six decades of major efforts in schistosomiasis control, substantial progress has been achieved. For example, the estimated number of schistosomiasis cases was reduced from 11.6 million in 1950s to 27,321 in 2024 [6, 7]. Thus, in accordance with the latest targets set by the World Health Organization, the Chinese central government proposed the goal of eliminating schistosomiasis transmission by 2030 [8]. Despite these progress and ambitious goals, several major challenges persist. In some impoverished regions, livestock farming, particularly cattle and water buffalo, serves as the primary income source for local farmers, while draft animals remain indispensable for agricultural production [9]. These socioeconomic factors significantly hinder the effective management of animal reservoirs of infection. Concurrently, environmental protection initiatives including pollution control and ecological restoration have inadvertently expanded suitable habitats for *O. hupensis* [3]. Moreover, the application of chemical molluscicides remains limited due to their adverse ecological effects.

Yunnan Province was previously a severe hotspot for *S. japonicum* endemism in China, with Dali and Eryuan counties around Erhai Lake having the most serious epidemics [10]. In recent years, an ecological environmental management program has been implemented in these endemic areas. After more than a decade of systematic conservation and management, significant achievements have been made in protecting and restoring the ecological environment of the basin. Water quality in Erhai Lake has markedly improved, with reduced levels of eutrophication. A comprehensive agricultural nonpoint source pollution prevention and control system has been established, achieving fully integrated operation of the sewage interception and treatment project system. Wetlands have been significantly restored. Relevant protection and governance measures have not only improved the water quality and ecological environment of the watershed, but also affected the transmission of *S. japonicum*. It is conducive to the control of *S. japonicum*, but it may also create a suitable breeding environment for snails, leading to an increase in the risk of epidemics. For example, the harmless treatment of the collected livestock feces, along with relocation or closure of livestock farms, facilitates the management of *S. japonicum* animal reservoirs and interrupts the transmission pathway of eggs into water. However, wetland restoration creates more favorable habitats for *O. hupensis* [11], which poses challenges for *S. japonicum* control efforts.

Given the ecological conservation significance of Erhai Lake and the goal of schistosomiasis elimination, it is necessary to propose strategies to harmonize ecological environment protection with schistosomiasis control. This study herein aimed to comprehensively analyze the impact of ecological environmental management program on *S. japonicum* control in Erhai Lake, and proposes strategic alternatives with a prioritized order of implementation using the SWOT-ANP-ADAM approach [12]. The strengths, weaknesses, opportunities, and threats (SWOT) analysis is conducted to identify the impact factors of ecological environmental management program on *S. japonicum* control and strategic alternatives. The Analytical Network Process (ANP) was used to evaluate the impact factors, and the Axial Distance-Based Aggregated Measurement (ADAM) method constructed multi-faceted polyhedron for the ranking of the strategic alternatives to better inform decisions for synergistic ecological-disease management. Our results may provide important evidence to improve integrated strategies for disease control in schistosomiasis endemic areas where conservation management programs are implemented.

## Methods

### Study area

Erhai Lake, the second largest plateau freshwater lake in Yunnan Province of China, is located within the watershed zone of the Lancang River, Jinsha River, and Yuanjiang River (100°05´–100°17´E, 25°36´–25°58´N) [13]. The lake basin covers an area of 2565.2 km^2^ with a 129 km shoreline. The region experiences a mean annual temperature of 15.1 °C, 2250–2480 h of annual sunshine, and receives 886 mm of average yearly precipitation. The socioeconomic activities of Erhai Lake region are mainly centered on agriculture, livestock farming, fisheries, and tourism [14]. Notably, the region is a serious *S. japonicum* endemic area.

In recent years, an ecological environmental management program has been implemented in the Erhai Lake basin, achieving significant improvements. The program focused on five key interventions [15–17]: (1) control of agricultural non-point source pollution across the lake basin, (2) water quality improvement of inflow rivers, (3) ecological rehabilitation of littoral zones, (4) construction of rural water supply and wastewater treatment facilities, and (5) establishment of a lake ecosystem management system. These interventions have not only improved the water quality of Erhai Lake, but have also enhanced the ecological environment around the basin [18].

### Information collection

Based on published information from literatures, books, and reports [19–28], this study analyzed the characteristics of ecological environmental management program in Erhai Lake and the schistosomiasis control program in Yunnan Province of China, and the endemic status of schistosomiasis in Yunnan Province of China.

**Fig. 1:**
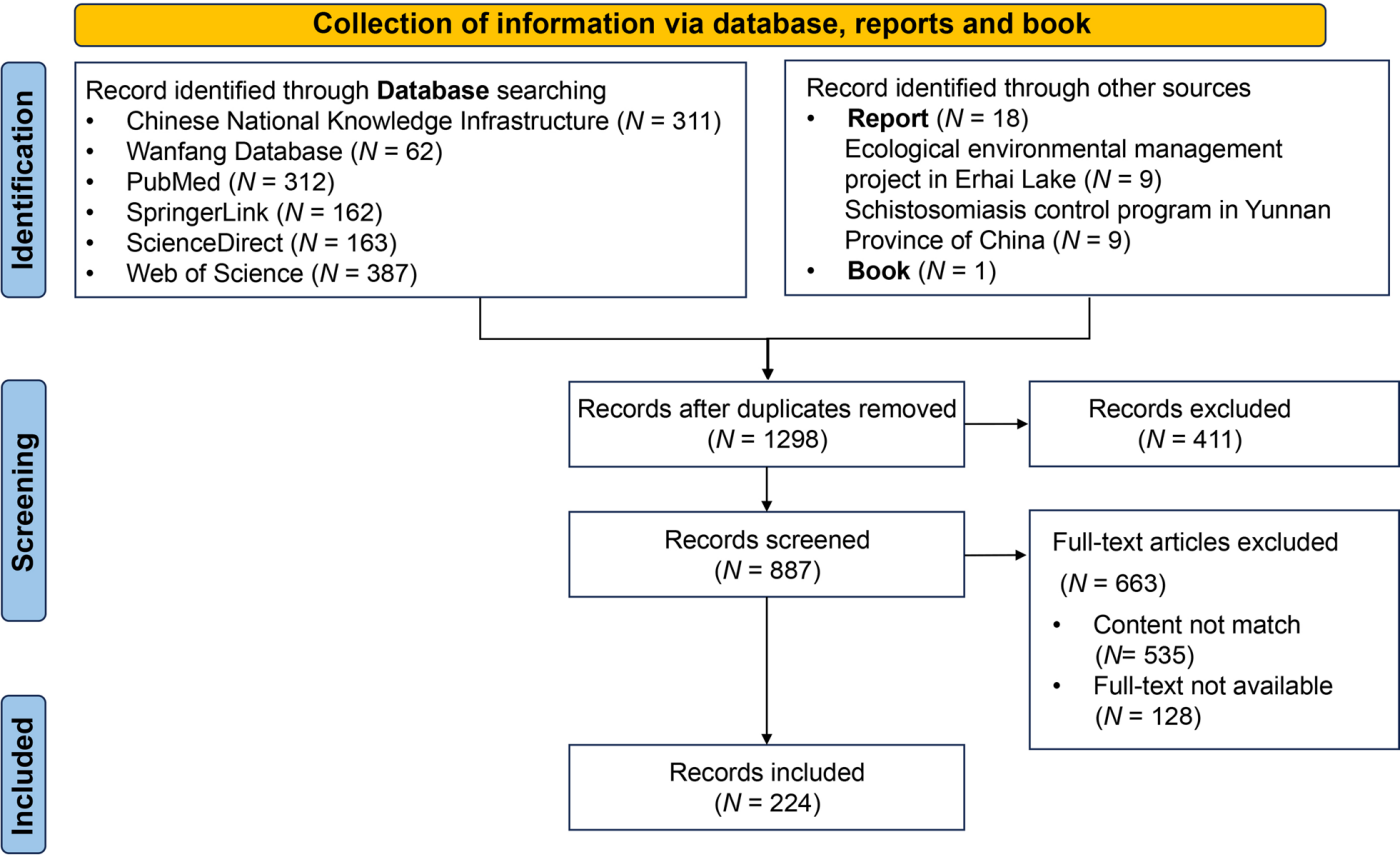
Flowchart of information collection

The flowchart of information collection is presented in Fig. 1. The information for this study was based on the 205 published literatures from 1990 to 2024 in the databases including Chinese National Knowledge Infrastructure (https://www.cnki.net/), Wanfang Database (https://c.wanfangdata.com.cn), Web of Science (https://www.webofscience.com/), PubMed (https://pubmed.ncbi.nlm.nih.gov/), Science Direct (https://www.sciencedirect.com/), and SpringerLink Database (https://link.springer.com/) and book related to ecological environmental management program in Erhai Lake. The key words for literatures search included schistosomiasis control, schistosomiasis prevention, environmental protection, ecological environmental management, and snail control. Meanwhile, this study also collected information from the reports of the ecological environmental management program in Erhai Lake and the schistosomiasis control program in Yunnan Province of China from 2016 to 2024. The characteristics of included information are presented in the Additional file 1.

### SWOT analysis

A skilled panel of researchers analyzes the impact factors (i.e., strengths, weaknesses, opportunities, and threats) of ecological environmental management program on schistosomiasis control. Then, we matched these factors with each other for system analysis to formulate strategic alternatives, including a pioneering strategy with strengths and opportunities (SO), a positive strategy with strengths and threats (ST), a conservative strategy with weaknesses and opportunities (WO), and a resistive strategy of weaknesses and threats (WT) [29, 30]. Meanwhile, we analyzed the characteristics of the relationships between internal (i.e., strengths and weaknesses) and external (i.e., opportunities and threats) factors, with focus on how to fully make use of strengths and opportunities and avoid weaknesses and threats, in order to formulate the integrated development strategic alternatives.

### ANP analysis

ANP analysis was used to obtain the weights of strengths, weaknesses, opportunities, and threats [31]. In ANP, the relations between groups (strengths, weaknesses, opportunities, challenges) and factors of each group (S1, …, Sn, W1, …, Wn, O1, …, On, T1, …, Tn) are hierarchical, and there is a bidirectional inner and outer relation between elements [12].

First, a panel of 60 experts in relevant fields was constructed, the characteristics of experts are presented in Table 1. The panel of expert recruitment criteria were: (1) In-depth knowledge of the ecological and environmental characteristics of Erhai Lake, the ecological environmental management program and the schistosomiasis epidemic within Erhai Lake basin; (2) Research or governance expertise, i.e., (1) Experts in administration and policy field have experienced in environmental protection or public health policy formulation, program management, or evaluation, (2) Experts in health, agriculture, forestry, and water conservancy of schistosomiasis control field have experienced in practical and academic research on schistosomiasis epidemiology and the relationship between environment and schistosomiasis, (3) Experts in environment and ecology field have experienced in practical and academic research on Erhai Lake ecological conservation, water environment management, wetland restoration, etc.; (3) Fluency in Mandarin, and willingness to participate in questionnaires and interviews; (4) No potential conflicts of interest that could affect the objectivity of the assessment and were anonymized during analysis process.

**Table 1:** Characteristics among 60 experts (*N*, %)

Characteristics		Sector					
		Administration and policy	Health of schistosomiasis control	Agriculture of schistosomiasis control	Forestry of schistosomiasis control	Water conservancy of schistosomiasis control	Environment and ecology
Years of experience	< 5	0 (0.0)	1 (10.0)	1 (10.0)	0 (0.0)	0 (0.0)	1(10.0)
	5‒15	3 (30.0)	3 (30.0)	4 (40.0)	6 (60.0)	7 (70.0)	4 (40.0)
	> 15	7 (70.0)	6 (60.0)	5 (50.0)	4 (40.0)	3 (30.0)	4 (40.0)
Professional qualifications	Primary	NA	1 (10.0)	2 (20.0)	2 (20.0)	1 (10.0)	1 (0.0)
	Intermediate	NA	4 (40.0)	4 (40.0)	4 (40.0)	5 (50.0)	4 (40.0)
	Senior	NA	5 (50.0)	4 (40.0)	4 (40.0)	4 (40.0)	4 (40.0)
Regional distribution	Erhai Lake basin	10 (100.0)	7 (70.0)	10 (100.0)	10 (100.0)	10 (100.0)	10 (100.0)
	Another region	0 (0.0)	3 (30.0)	0 (0.0)	0 (0.0)	0 (0.0)	0 (0.0)

*NA* Not applicable or not available

Second, the existing dependencies between each factor are identified through SWOT analysis. These network relations allow for the building of the influential super matrix, which has a dimension of 14 × 14 (i.e., 14 rows and 14 columns, each corresponding to the 14 identified impact factors). Each element m_ij_ of this matrix is filled with 1 or 0 values, where 1 means that the i-th factor is influenced by j-th factor [12].

Third, the experts expressed the influence of each factor over every other one by using Saaty’s scale (from 1 = “equally important” to 9 = “enormously more important”) [31], with higher scores indicating greater importance. The experts’ evaluations were statistically merged using a probability distribution, with the most frequent assessments representing the entire focus group [32]. Based on the results of the expert’s evaluations, an unweighted super matrix was established, and a weighted super matrix was established by the weight of each factor is multiplied by the weight of each group.

Fourth, a limiting super matrix was established and the factors weights was obtained, the desired factors weight that allow for their final rating. Higher value of weights represents the more important of factors.

ANP analysis was conducted using Super Decision (version 3.2) software (Creative Decisions Foundation, Pittsburgh, PA, USA).

### ADAM analysis

ADAM analysis was used to obtain the prioritized implementation sequence of strategic alternatives [33]. The ADAM method represents an entirely new group of Multicriteria decision making (MCDM) methods [34].

First, six criteria for evaluating the strategic alternativeswere defined based on literature review and the opinions of the previously established 60 expert panel. These criteria emerged from panel discussions evaluating potential standards for importance and operational feasibility, which considered the potential criteria in the discussion groups, including high cost-effectiveness ratio (C1), human health promotion (C2), animal health promotion (C3), ecological balance maintenance (C4), potential environmental negative effects (C5), and extrapolation feasibility (C6).

Second, the weight of six criteria was obtained by ANP analysis, as described in the preceding section.

Third, the expert panel evaluated the strategic alternatives ' compliance using a nine-point scale (from 1 = “none” to 9 = “Extremely High”), with higher scores indicating greater degrees of compliance. The experts’ evaluations were statistically merged using a probability distribution, with the most frequent assessments representing the entire focus group [32].

Fourth, the sequence of the importance of strategic alternatives by computing the volumes of complex polyhedron constructed within a three-dimensional coordinate system [34], with higher volumes indicating the more important of strategic alternatives. Meanwhile, to enhance the interpretability and decision-readiness of these results, we further propose an operational tiering system (i.e., short-, medium-, and long-term actions) and calculate the relative advantage percentage for each strategy. This metric expresses each strategy's composite score as a percentage of the top-ranked strategy's score (set at 100%).

Fifth, sensitivity analysis was used to check the stability of the obtained ranking strategic alternatives [34]. A total of 21 scenarios were defined, each involving a change in the weights of the three most important criteria, the weight of each criterion was reduced by 15%, 30%, 45%, 60%, 75%, 90%, and 100%, respectively. We then calculated Spearman's rank correlation (*r*_s_) between the ranking 21 scenarios and the initial ranking, and tested their significance.

ADAM analysis was conducted using ADAM software package developed by Krstić and Kovač (http://adam-mcdm.com/). The Spearman's rank correlation analysis using IBM SPSS Statistics 26.0 (IBM, Armonk, NY, USA), the statistical tests were two-sided, with a *P* value less than 0.05 considered statistically significant.

## Results

### SWOT results

A total of 14 impact factors of ecological environmental management program on schistosomiasis control in Erhai Lake, as well as 12 strategic alternatives were obtained by SWOT analysis.

#### Strength factors

The implementation of high-efficiency water-saving irrigation systems has created unfavorable habitats for the *O. hupensis*, thereby contributing to schistosomiasis control (S1). The high-efficiency water-saving irrigation systems have replaced traditional irrigation methods, which previously maintained prolonged waterlogging in farmlands. Such waterlogged conditions provided favorable environments for habitats of *O. hupensis*, while simultaneously facilitating wider dispersion of schistosome cercariae in water bodies.

Management of agricultural non-point source pollution contributes to *S. japonicum* animal reservoirs control (S2). The promotion of green fertilizer as alternative to livestock feces-based fertilizer for crop cultivation, and the harmless treatment of the collected livestock feces, along with relocation or closure of livestock farms, which can help to interrupt the transmission pathway of eggs into water.

Construction of the water supply infrastructure can minimize human exposure to water infested cercariae, thereby reducing *S. japonicum* transmission risks (S3).

Rural land transfer initiative has changed agricultural fields into commercially managed zones, significantly reducing habitats for *O. hupensis *(S4). Through transitioning local economies from agriculture and livestock farming to commercial activities, this program has reduced the risk of *S. japonicum* transmission by decreasing the frequency of residents and livestock contact with water containing cercariae.

#### Weakness factors

Wetland construction creates more favorable habitats for *O. hupensis*, as well as increases the number of wildlife populations in hilly areas, posing challenges for schistosomiasis control (W1). After wetland construction, the lush vegetation and humid microclimate provide favorable conditions for snails breeding, potentially leading to the emergence and re-emergence habitats of *O. hupensis*.

During rural land transfer processes, temporarily unused plots with overgrown vegetation create favorable habitats for *O. hupensis*; at the same time, some idle lands remain inaccessible for *S. japonicum* control interventions, elevating transmission risks (W2).

In ecological environment protection areas, the chemical molluscicides such as niclosamide has been limited, because the usage of fertilizers and pesticides containing nitrogen and phosphorus has been enhanced regulation (W3).

The transplantation of vegetation from persistent habitats areas of *O. hupensis* during wetland construction may introduce snails via soil and plant material, thereby facilitating their dispersal and increasing *S. japonicum* transmission risks (W4).

#### Opportunity factors

The One Health and green development concepts provide innovative perspectives and opportunities for *S. japonicum* control (O1). Collaborative efforts between disease control and ecological conservation departments can implement comprehensive interventions to achieve coordinated progress in both environmental protection and *S. japonicum* elimination.

Long-term planning and sustained funding for ecological environmental management program ensure the continuity and effectiveness of schistosomiasis control interventions integrated within these initiatives (O2).

The Chinese central government's prioritization of *S. japonicum* control and ecological environmental management has provided policy and financial for developing synergistic ecological-disease management that concurrently advances environmental conservation and disease elimination objectives (O3).

#### Threat factors

The lack of a top-level cooperative mechanism designed to integrate *S. japonicum* control and ecological environmental management may lead to uncoordinated efforts between ecological environmental management and disease control measures, leading to inefficient resource utilization (T1).

The balance of ecological benefits with economic, social, and *S. japonicum* control is essential; otherwise, governance measures may become one-sided and unsustainable (T2).

Certain *S. japonicum* control interventions may adversely impact biodiversity, creating conflicts with the objectives of ecological environmental management program (T3). For example, *O. hupensis* control measures (e.g., channel consolidation and bank hardening) may weaken ecological connectivity between watercourses and riparian ecosystems, disrupt biological corridors between different communities created by bank delineation, and ultimately reduce the biodiversity of riverine lake wetland systems.

#### Strategic alternatives

Construction of the ecological management-*S. japonicum* control model in an integrated manner (SO1). Implementing *S. japonicum* control and ecological management measures based on the *S. japonicum* epidemic characteristics and environmental conditions of regions, following the principles of localization and precision (SO2). Allocation of special funds for the ecological management-*S. japonicum* control model (SO3).

Delineate the different construction areas of the ecological management- *S. japonicum* control model, including key construction areas, optimization and upgrading areas, reasonable control areas, and prohibited construction areas (ST1). Promoting the harmonization of social, economic, and ecological benefits (ST2). Accelerating the development of rapid, simple, and sensitive *S. japonicum* detection technologies and environmentally friendly molluscicide (ST3). Continuously evaluate the quality and effectiveness of ecological management measures in terms of *S. japonicum* control effects (ST4).

Strengthen efforts of *O. hupensis* monitoring, and improve monitoring sensitivity (WO1). Carrying out monitoring of foreign populations and wild mammals (WO2). Regularization of health education on prevention of *S. japonicum* (WO3).

Adjusting the scope of application of ecological environmental management and *S. japonicum* control measures in view of their shortcomings (WT1). Changing the *O. hupensis* control strategy from comprehensive eradication to a certain degree of control (WT2).

### ANP results

Weights and ranking of groups of impact factors obtained by the ANP analysis are presented in Table 2. The relative importance of the group is ranked as strengths (weight equal to 0.3092), opportunities (weight equal to 0.2610), weaknesses (weight equal to 0.2324), and threats (weight equal to 0.1975).

**Table 2:** Weights and ranking of groups obtained by the ANP analysis

Group	Weights	Rank
Strengths	0.3151	1
Weakness	0.2264	3
Opportunities	0.2610	2
Threats	0.1975	4

Ecological environmental management project in the Erhai Lake has significant strengths and opportunities in promoting *Schistosoma japonicum* control, but also faces certain weaknesses and threats

*ANP* Analytical network process

Weights and ranking of impact factors obtained by the ANP analysis are presented in Table 3. The top five factors are ranked as O3 (weight equal to 0.1562), T1 (weight equal to 0.1424), S2 (weight equal to 0.1209), S3 (weight equal to 0.0853), W1 (weight equal to 0.0700).

**Table 3:** Weights and ranking of factors obtained by the ANP analysis

Factor	Weights	Rank
S1	0.0668	6
S2	0.1209	3
S3	0.0853	4
S4	0.0421	11
W1	0.0700	5
W2	0.0332	13
W3	0.0624	7
W4	0.0608	8
O1	0.0607	9
O2	0.0441	10
O3	0.1562	1
T1	0.1424	2
T2	0.0335	12
T3	0.0217	14

*S* Strengths, *W* Weakness, *O* Opportunities, *T* Threats, *ANP* Analytical Network Process

### ADAM results

**Fig. 2:**
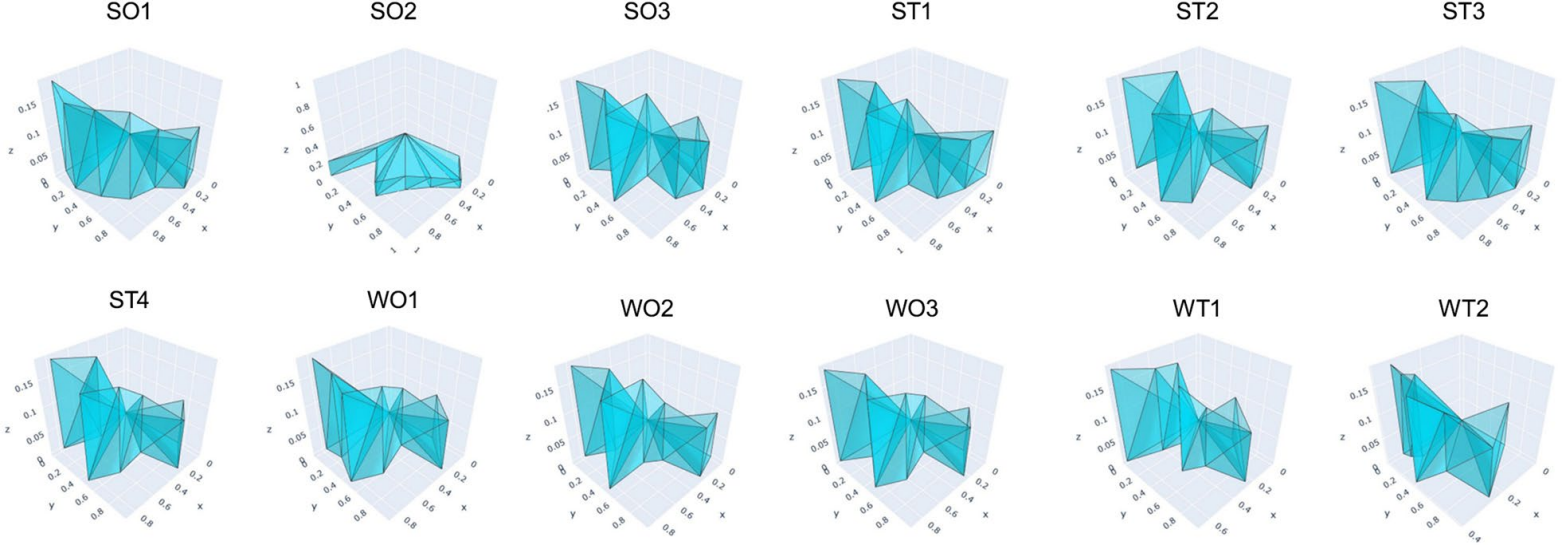
Complex polyhedron for the Strategic alternatives. The higher volumes of complex polyhedron that are defined by points (vertices) in a three-dimensional coordinate system, indicating higher prioritized implementation sequence. Each point belongs to one of three types: coordinate origin (O), reference point (R), and weighted reference point (P). The coordinate origin (O) is the point with coordinates (0,0,0). Reference points (R) have coordinates (x, y, 0), where the axial distance from the origin on the x–y plane represents the scheme's score under a specific criterion. Weighted reference points (P) have coordinates (x, y, z), where the z-coordinate calculates the axial distance from the x–y plane. These distances values correspond to the weights assigned to each criterion

Complex polyhedron for 12 strategic alternatives is presented in Fig. 2, and volumes of the complex polyhedron and ranking of the strategic alternatives are presented in Table 4, the volume reflects integrated multi-dimensional benefits.

**Table 4:** Ranking, relative advantage percentages and operational tiering of the strategic alternatives

Strategies alternative	Volume	Rank	Relative advantage percentages (% of best)	Operational tiering^#^
SO1	0.0693	1	100.00	C
SO2	0.0540	3	77.92	A
SO3	0.0503	5	72.60	C
ST1	0.0536	4	77.35	C
ST2	0.0429	10	61.88	C
ST3	0.0594	2	85.77	A
ST4	0.0446	8	64.42	B
WO1	0.0499	6	72.09	A
WO2	0.0435	9	62.86	A
WO3	0.0492	7	71.05	A
WT1	0.0260	11	37.57	B
WT2	0.0205	12	29.58	B

*SO* strengths and opportunities, *ST* strengths and threats, *WO* weaknesses and opportunities, *WT* weaknesses and threats

^#^Operational tiering A is short-term action, operational tiering B is medium-term action, operational tiering C is long-term action

The strategic alternatives priorities for implementation are, in order of priority, SO1, ST, SO, ST1, SO, WO1, WO3, ST4, WO2, ST, WT1, and WT2, with volume of complex polyhedron equal to 0.0693, 0.0594, 0.0540, 0.0536, 0.0503, 0.0499, 0.0492, 0.0446, 0.0435, 0.0429, 0.0260, 0.0205, respectively.

Meanwhile, we propose an operational tiering system (A/B/C). Tier A (short-term actions) includes SO2, ST3, WO1, WO2, WO3, with relative advantage percentages equal to 77.92%, 85.77%, 72.09%, 62,85%, and 71.05%, respectively. Tier B (medium-term actions) includes ST4, WT1, WT2, with relative advantage percentages equal to 64.42%, 37.57%, and 29.58%, respectively. Tier C (long-term actions) includes SO1, SO3, ST1, ST2, with relative advantage percentages equal to 100.00%, 72.60%, 77.35%, and 61.88%, respectively.

Furthermore, the details of the sensitivity analysis are presented in the Additional file 2. Based on the results of the sensitivity analysis, in all scenarios, the ranking of WT1 (ranked eleventh) and WT2 (ranked twelfth) were unchanged, other strategic alternatives slightly changed their rank throughout the scenarios. The values of *r*_s_ ranged between 0.895 and 0.993 (*P*_all_ < 0.05), and the average value for all scenarios was 0.954, it can be concluded that there are no significant changes in the obtained rankings. Therefore, it can be concluded that the obtained ranking of strategic alternatives is stable enough.

## Discussion

Eliminating schistosomiasis remains a challenging public health activity worldwide [35]. The *S. japonicum* transmission is closely related to the natural environment and socio-economic factors [36], consequently, changes in the surrounding environment will inevitably affect the *S. japonicum* epidemiology. The ecological environmental management program of Erhai Lake has become a model for lake management in China [37]. This initiative has achieved remarkable results, significantly improving both water quality in Erhai Lake and the broader watershed ecosystem [38]. It is very important to control *S. japonicum* in combination with ecological environmental management program. To our knowledge, this is the first application of the integrated SWOT-ANP-ADAM approach to develop and prioritize strategies for schistosomiasis control in the context of ecological management. While previous studies have employed MCDM methods or One Health concepts in public health, our work uniquely combines SWOT, ANP, and ADAM to evaluate the interplay between ecological environmental management program and disease control, offering a novel and actionable framework for integrated environmental-health management.

In this study, we first used SWOT analysis to obtain the strengths, weaknesses, opportunities, and threat factors of the ecological environmental protection programs on schistosomiasis control in Erhai Lake, and then obtained the weights and rankings of these factors by ANP analysis. The results emphasized that the relative importance is ranked in the order of strengths, opportunities, weaknesses, and threats. This implies that *S. japonicum* control is positively influenced by ecological environmental management program in Erhai Lake basin, and the ecological environmental management program has significant strengths and opportunities in promoting *S. japonicum* control, but also faces certain weaknesses and threats. The foregoing results are consistent with other previous study performing system modelling in Eryuan County of Erhai Lake basin, this study suggests that ecological approaches implemented in schistosomiasis endemic areas are able to improve the co-effectiveness of environmental protection and schistosomiasis control, providing a new avenue for eliminating schistosomiasis thanks to the application of precise interventions [39].

Among all the influencing factors, O3 and T1 received the highest weights. The results indicate that a top-level cooperative mechanism for integrating ecological environmental protection and *S. japonicum* control has not yet been established; however, the Chinese central government's emphasis on ecological environmental protection and *S. japonicum* control provides policy and financial support for further integrating resources and constructing synergistic ecological-disease management.

Several other factors also received relatively high weights, including S2, S3, W1, S1, and W3. The impact of relevant interventions implementation in ecological environmental management program on schistosome transmission is of concern. Management of agricultural non-point source pollution, construction of high-efficiency water-saving irrigation systems, and implementation of high-efficiency water-saving irrigation systems reduce the risk of *S. japonicum* transmission in Erhai Lake.

First, the transmission of *S. japonicum* requires that feces containing parasite eggs from infected cattle, pigs, and other infectious sources enter water [40]. Fecal management has long been proven to be one of the effective ways to control *S. japonicum* in China [41]. In the Erhai Lake, to control agricultural non-point source pollution, the program promotes the use of green fertilizers as a substitute for fertilizers derived from livestock feces. This substitution prevents feces containing *S. japonicum* eggs from being applied to agricultural land, thereby interrupting a potential pathway for egg contamination of water. In addition, the program has formulated the relevant policies to determine the scope of the basin's livestock no-farming zone, restricted-farming zone and permissible-farming zone, and has shut down and relocated many large-scale farms [42], which has resulted in a significant reduction in the number of cattle, pigs and other sources of *S. japonicum* infection. Notably, after collecting livestock feces from the entire Erhai Lake basin, local organic fertilizer processors use the high-temperature, aerobic composting and fermentation process to produce ecological organic fertilizer based on the “Shunfeng Erhai model” [15], which can effectively kill pathogens such as *S. japonicum* eggs in livestock feces.

Second, in order to reduce direct use of lake waterby residents for production and living, the ecological environment protection program has carried out the construction of water supply infrastructure, including piped water to households, centralized water supply station, safe wells, etc.. [43]. These interventions provide clean and safe water to residents in *S. japonicum* endemic areas [37], substantially reducing the frequency of residents' contact with water containing cercariae for drinking, washing, irrigation, and other needs has reduced, which reduces the risk of *S. japonicum* transmission.

Third, *O. hupensis* is the sole intermediate host of *S. japonicum* and thus plays a key role in disease transmission [44]. Yunnan Province is the hilly schistosomiasis endemic area, and *O. hupensis* mainly harbor in irrigation ditches, field ridges, wasteland, and mud fields, and prefers to survive in dark and moist environments [45]. Within ecological protection and management Program of Erhai Lake, the implementation of high-efficiency water-saving irrigation systems, including the improvement of water intake gates, cisterns, and field diversion pipelines [15], has had important implications for *O. hupensis* control. The traditional crops’ natural irrigation method was replaced, which resulted in long-term flooding of farmland and the formation of large areas of stagnant water, which is extremely favorable for the survival and reproduction of *O. hupensis*.

While, the wetland restoration may create more favorable habitats for *O. hupensis* and lead to an increase in the number of wild animals, as well as the use of chemical molluscicides has been limited in ecological environment protection areas, posing challenges for *S. japonicum* control. Previous studies have shown that the wetland environment is suitable for the growth and reproduction of *O. hupensis* [46]. In 2023, 40,000 acres of wetlands were constructed in Erhai Lake basin [15]. As the gradual improvement of the natural environment after the construction of the wetland, lush vegetation and humid microclimate may result the emergence and re-emergence habitats of *O. hupensis* [37]. Indeed, monitoring data indicate that the average density of live *O. hupensis* has increased from 0.0226 per frame in 2016 before construction to 0.0685 per frame in 2024. In addition, wild animals (e.g., rodents) play an important role in *S. japonicum* transmission [47]. The increase in the number and range of activities of wild animals after the wetland restoration poses a potential risk for the *S. japonicum* control. Normal chemical molluscicides are toxic to aquatic animals and cause environmental pollution [48]. Due to ecological environmental protection requirements in Erhai Lake basin, the use of chemical molluscicides, the most effective means of *O. hupensis* control [3], has been limited in some areas.

In addition, using ADAM analysis to calculate the complex polyhedron volumes, we obtained the prioritized order of strategic alternatives for integrating ecological environmental protection with schistosomiasis control. To enhance practical applicability, we further proposed an operational tiering system aligned with short-, medium-, and long-term actions. Short-term actions involve implementing schistosomiasis control and ecological management measures tailored to local epidemiological characteristics and environmental conditions. Concurrently, development or introduction of rapid, simple, and sensitive *S. japonicum* detection technologies, and environmentally friendly molluscicide. Monitoring of *O. hupensis*, foreign populations, and wildlife must be strengthened, while ensuring regular health education to prevent *S. japonicum* transmission. Medium-term actions involve conducting the quality and effectiveness evaluation of ecological management measures in relation to their impact *S. japonicum* control, and adjusting the scope and intensity of interventions based on identified shortcomings. Meanwhile, shift the *O. hupensis* control strategy from comprehensive eradication to a certain degree of control, balancing disease control requirements with ecological environmental management. Long-term actions focus on establishing an ecological management- *S. japonicum* control model. Dedicated funding will be allocated for its implementation across four distinct zones (i.e., key construction areas, optimization and upgrading areas, reasonable control areas, and prohibited construction areas), promoting the integration of social, economic, and ecological benefits.

Furthermore, our study showed that construction of the ecological management-*S. japonicum* control model in an integrated manner is the most important strategy [39]. It is also important to implement *S. japonicum* control and ecological management measures based on the epidemic characteristics and environmental conditions of regions, following the principles of localization and precision [49, 50]. At the same time, we should also accelerate the development of rapid, simple, and sensitive schistosomiasis detection technologies and environmentally friendly molluscicides. Such as, a novel plant molluscicide, tea-seed distilled saponins, has shown to be lethal to snails and less toxicity to other organisms [51, 52].

However, there is limitation to this study. During information collection, our literature search was restricted to Chinese and English language publications. This language limitation may have resulted in the omission of relevant evidence published in other languages. Additionally, during ANP and ADAM analysis, we statistically aggregated the evaluation results from the 60-person expert panel using probability distribution. The most frequently occurring evaluation result represented the consensus opinion of the entire focus group. While this method reduces the influence of outlier opinions, it introduces a degree of subjectivity and may not fully capture the diversity of expert perspectives. Future research may employ the Delphi method or larger-scale surveys to further refine the results of this study [53].

## Conclusions

Our results indicate that ecological environmental management program in Erhai Lake basin has significant strengths and opportunities in promoting *schistosomiasis* control, but also faces certain weaknesses and threats. The Chinese central government's prioritization of *S. japonicum* control and ecological environmental management, as well as the lack of a top-level cooperative mechanism designed to integrate *S. japonicum* control and ecological environmental management, are the more noteworthy factors. Secondly, in ecological environmental management program of Erhai Lake, management of agricultural non-point source pollution, construction of high-efficiency water-saving irrigation systems, and implementation of high-efficiency water-saving irrigation systems reduce the risk of *S. japonicum* transmission in the local area, while the wetland restoration may create more favorable habitats for *O. hupensis*, as well as increase the number of wildlife populations posing challenges for *S. japonicum* control.

Additionally, 12 strategic alternatives with prioritized implementation may provide suggestions for decision makers in similar areas to adopt strategic decisions, which are of great practical significance and application value.

## Supplementary Information

Additional file 1.

Additional file 2.

## Data Availability

The datasets used and/or analyzed during the current study can be accessed with permissions from Yunnan Institute of Endemic Disease Control & Prevention and contact Yi Dong.

## Abbreviations

SWOT: Strengths, weaknesses, opportunities, and threats
ANP: Analytical Network Process
ADAM: Axial Distance-Based Aggregated Measurement
SO: Strengths and opportunities
ST: Strengths and threats
WO: Weaknesses and opportunities
WT: Weaknesses and threats
MCDM: Multicriteria decision making
