# Supplementary material for: Ecological environmental management program promotes Schistosomiasis control in Erhai Lake of China: an analysis based on SWOT-ANP-ADAM approach
Source: Infect Dis Poverty. 2026 Apr 13;15:44. doi: 10.1186/s40249-026-01442-9 (PMC13072597; doi:10.1186/s40249-026-01442-9)
Supplement: Supplementary file 2 — Additional file 2. [file 40249_2026_1442_MOESM2_ESM.pdf]

Sensitivity analysis

In Axial Distance-Based Aggregated Measurement (ADAM) analysis, sensitivity analysis aims to assess how changes in the relative weights of criteria used in the decision-making process affect the final outcomes and rankings of strategic alternatives.

In this study, a total of 21 scenarios were defined, each involving a change in the weights of the three most important criteria (**eTable1**). In the first seven scenarios (Sc.0-Sc.7), the weight of the most important criterion (C2, i.e., human health promotion) was reduced by 15%, 30%, 45%, 60%, 75%, 90%, and 100%, respectively. Concurrently, the weights of all other criteria were proportionally scaled to give a sum of 1. The remaining 14 scenarios (Sc.8-Sc.21) were formed in the same way, by reducing the weights of the second (C1, i.e., high cost-effectiveness ratio) and third (C7, i.e., extrapolation feasibility) most important criteria and scaling the remaining ones.

eTable1 Criteria weights in sensitivity analysis scenarios

	C1	C2	C3	C4	C5	C6	C7
Sc.0	0.166667	0.180399	0.095593	0.122130	0.101878	0.166665	0.166668
Sc.1	0.179670	0.153339	0.103040	0.131658	0.109825	0.179667	0.179670
Sc.2	0.192672	0.126280	0.110488	0.141186	0.117772	0.192668	0.192673
Sc.3	0.205674	0.099220	0.117935	0.150715	0.125720	0.205670	0.205676
Sc.4	0.218677	0.072160	0.125383	0.160243	0.133667	0.218671	0.218679
Sc.5	0.231679	0.045100	0.132830	0.169771	0.141614	0.231673	0.231682
Sc.6	0.244681	0.018040	0.140278	0.179300	0.149562	0.244674	0.244685
Sc.7	0.249395	0.000000	0.142932	0.182748	0.152467	0.249388	0.249394
Sc.8	0.141667	0.214938	0.113864	0.145456	0.121368	0.189658	0.189660
Sc.9	0.116667	0.249477	0.132135	0.168783	0.140857	0.212524	0.212527
Sc.10	0.091667	0.284016	0.150406	0.192109	0.160346	0.235391	0.235394
Sc.11	0.066667	0.318555	0.168677	0.215436	0.179835	0.258257	0.258260
Sc.12	0.041667	0.353094	0.186948	0.238762	0.199324	0.281124	0.281127
Sc.13	0.016667	0.387633	0.205219	0.262088	0.218813	0.303990	0.303994
Sc.14	0.000000	0.401193	0.212177	0.271079	0.226175	0.314384	0.314388
Sc.15	0.202335	0.219083	0.098465	0.148272	0.123690	0.202333	0.141668
Sc.16	0.238004	0.257716	0.115841	0.174438	0.145518	0.238001	0.116668
Sc.17	0.273673	0.296349	0.133218	0.200604	0.167347	0.273670	0.091668
Sc.18	0.309342	0.334982	0.150595	0.226769	0.189175	0.309338	0.066668
Sc.19	0.345011	0.373615	0.167971	0.252935	0.211003	0.345007	0.041668
Sc.20	0.380680	0.412248	0.185348	0.279101	0.232831	0.380675	0.016668
Sc.21	0.416349	0.450881	0.202724	0.305267	0.254660	0.416344	0.000000

Abbreviations: Sc scenario; C Criteria.

The ranking of strategic alternatives in different scenarios obtained by the ADAM analysis are presented in **eTable 2**, the changes in the ranking of strategic alternatives across different scenarios are presented in **eFigure 1**.

Based on the results of the sensitivity analysis, it can be seen that in all scenarios, the ranking of WT1 (ranked eleventh) and WT2 (ranked twelfth) were unchanged. Other strategic alternatives slightly changed their rank throughout the scenarios.

In addition, we calculated Spearman's rank correlation (r_s) of ranking Sc.1-Sc.21 with ranking Sc.0, and tested their significance using IBM SPSS Statistics 26.0 (IBM, Armonk, NY, USA). The statistical tests were two-sided, with a P value less than 0.05 considered statistically significant. In all scenarios, the values of r_s ranged between 0.895 and 0.993 ($P_{all}<0.05$), and the average value for all scenarios was 0.954, it can be concluded there are no significant changes in the obtained rankings.

Therefore, it can be concluded that the ranking obtained in the basic scenario is stable enough.

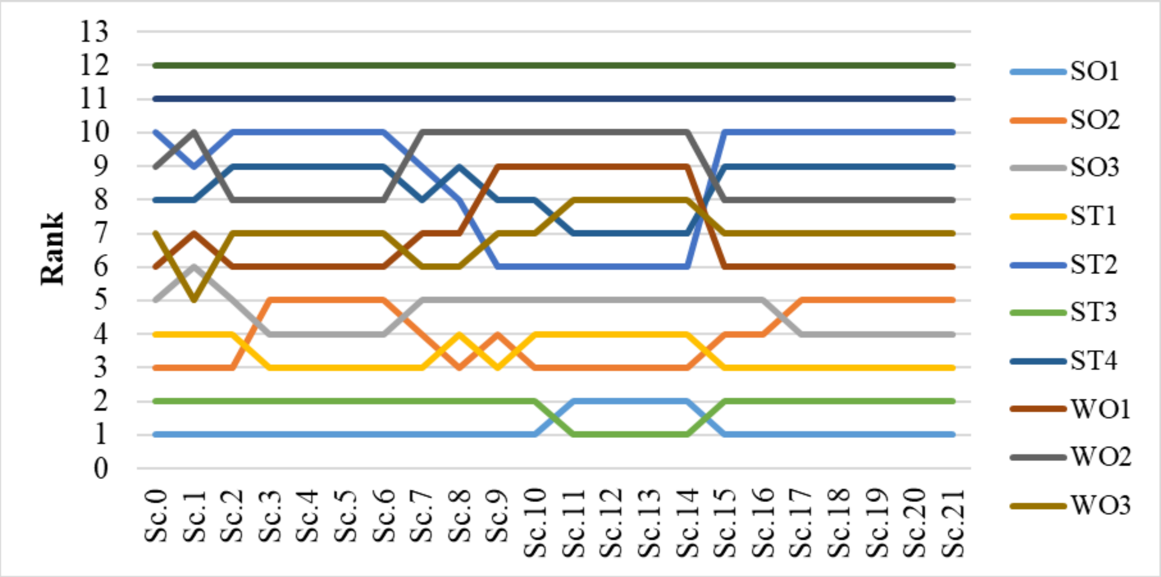

eFigure1 Changes in the ranking of strategic alternatives across different scenarios

eTable2 Ranking results obtained in different scenarios

	SO1	SO2	SO3	ST1	ST2	ST3	ST4	WO1	WO2	WO3	WT1	WT2	r_s	*P value*
Sc.0	1	3	5	4	10	2	8	6	9	7	11	12	NA	NA
Sc.1	1	3	6	4	9	2	8	7	10	5	11	12	0.972	<0.001
Sc.2	1	3	5	4	10	2	9	6	8	7	11	12	0.993	<0.001
Sc.3	1	5	4	3	10	2	9	6	8	7	11	12	0.972	<0.001
Sc.4	1	5	4	3	10	2	9	6	8	7	11	12	0.972	<0.001
Sc.5	1	5	4	3	10	2	9	6	8	7	11	12	0.972	<0.001
Sc.6	1	5	4	3	10	2	9	6	8	7	11	12	0.972	<0.001
Sc.7	1	4	5	3	9	2	8	7	10	6	11	12	0.979	<0.001
Sc.8	1	3	5	4	8	2	9	7	10	6	11	12	0.972	<0.001
Sc.9	1	4	5	3	6	2	8	9	10	7	11	12	0.902	<0.001
Sc.10	1	3	5	4	6	2	8	9	10	7	11	12	0.909	<0.001
Sc.11	2	3	5	4	6	1	7	9	10	8	11	12	0.895	<0.001
Sc.12	2	3	5	4	6	1	7	9	10	8	11	12	0.895	<0.001
Sc.13	2	3	5	4	6	1	7	9	10	8	11	12	0.895	<0.001
Sc.14	2	3	5	4	6	1	7	9	10	8	11	12	0.895	<0.001
Sc.15	1	4	5	3	10	2	9	6	8	7	11	12	0.986	<0.001
Sc.16	1	4	5	3	10	2	9	6	8	7	11	12	0.986	<0.001
Sc.17	1	5	4	3	10	2	9	6	8	7	11	12	0.972	<0.001
Sc.18	1	5	4	3	10	2	9	6	8	7	11	12	0.972	<0.001
Sc.19	1	5	4	3	10	2	9	6	8	7	11	12	0.972	<0.001
Sc.20	1	5	4	3	10	2	9	6	8	7	11	12	0.972	<0.001
Sc.21	1	5	4	3	10	2	9	6	8	7	11	12	0.972	<0.001

Abbreviations: NA Not applicable or not available; Sc scenario; SO strengths and opportunities; ST strengths and threats; WO weaknesses and opportunities; WT weaknesses and threats.
